# Cyberbullying and Empathy in the Age of Hyperconnection: An Interdisciplinary Approach

**DOI:** 10.3389/fsoc.2020.551881

**Published:** 2020-10-16

**Authors:** Vincenzo Auriemma, Gennaro Iorio, Geraldina Roberti, Rosalba Morese

**Affiliations:** ^1^Department of Political and Social Studies, Sociology, Università Degli Studi di Salerno, Fisciano, Italy; ^2^Department of Human Sciences, Sociology of Cultural and Communication Processes, University of L'Aquila, L'Aquila, Italy; ^3^Institute of Public Health, Faculty of Biomedical Sciences, Università della Svizzera Italiana, Lugano, Switzerland; ^4^Faculty of Communication, Culture and Society, Università della Svizzera Italiana, Lugano, Switzerland

**Keywords:** bullying, cyberbullying, empathy, hyperconnection, individualization, theory of mind

## Abstract

Considering cyberbullying as a challenging frontier of analysis in the social sciences, we find ourselves today with the duty to analyze it within a much broader social context. Indeed, we must take into account the logic of exclusion, as a fact. Today, in the logic of how the Internet works, a thin line separates the victim from the perpetrator; this is also due to the Internet we know today, made up of a mass and a headless power. Trying to amplify this dichotomy, we can say that today we live in the era of the so-called “ban-opticon” (or the logic of prohibition). This logic ranges from simply removing Facebook friends from the list, to excluding sources of knowledge. This article has focused on the discussion of cyberbullying by applying an interdisciplinary approach from sociology to psychology, with the analysis of important aspects such as empathy, hyperconnection, individualization. The concept of empathy, studied several times through the terms Verstehen and Einfuhlung, has today been explored by many parties. In fact, the term Empathy has been used to describe sympathy or compassion. The interdisciplinary approach allows a broader and more innovative analysis to better understand the phenomenon of cyberbullying and to conceptualize new intervention strategies in the social and educational fields to open new frontiers in research.

## Introduction

In the context of contemporary society, the need to identify new interpretative categories through which reading the complexity of the present is increasingly coming out. The broader aim of social researchers is developing adequate analytical tools and explanatory criteria suitable for re-defining the meaning of social action, fitting it into a multidisciplinary theoretical framework that overcomes the existing fences between the different fields of study. In this perspective, both sociology and neuroscience can offer a valuable contribution for interpreting the complexity of social ties and the dynamics of building subjects' identity, providing new tools through which analyzing innovative forms of social interactions.

Therefore, the proposed contribution aims to analyze the phenomenon of cyberbullying through a fully interdisciplinary approach, joining the attention to the fundamental aspects of social dynamics with an in-depth analysis of the role of physiological reactions related to emotional states.

In this perspective, the first part of the paper will aim at circumscribing the investigated phenomenon, identifying similarities and differences with respect to the most common forms of bullying; subsequently, starting from the considerations of authors such as Putnam and Bourdieu on the centrality of social capital in building of a community feeling, it will be highlight the role played by the dynamics of individualization in the process of deterioration of the subjects' social capital and how this can be interrelated with the spread of forms of cyberbullying. The second section of the article will focus on the concept of empathy identifying, starting from Singer and Lamm (2009), Lipps (1903), Berrios (2014), Pinotti (2014), Lamm et al. (2019) observations, a psychological model for understanding cyberbullying and its individual/social implications. Finally, the last part of the paper will insert cyberbullying in a wider sociological perspective, tracing in the idea of representation proposed by Goffman one of the most suggestive metaphors to frame this complex phenomenon.

## Cyberbullying: Essential Characterization

“A person is bullied when he or she is exposed, repeatedly and over time, to negative actions on the part of one or more other persons, and he or she has difficulty defending himself or herself” (Olweus, 1993, p. 78). A synthetic and effective definition of bullying is that of Sharp and Smith that speaks of “peer abuse,” that is a kind of social relationship between friends based on power and control roles. This phenomenon is characterized by aggressive behavior repeated over time. Shelley and Swearer (Olweus, 1978) underlined that the pioneering contributions of Olweus (Olweus, 1994; Endresen and Olweus, 2001; Katz, 2012; Vilella and Reddivari, 2020) have allowed to define this social problem as a subcategory of interpersonal aggression characterized by intentionality, repetition and an imbalance of power, distinguishing bullying from other forms of violence (Smith and Sharp, 1994; Smith and Myron-Wilson, 1998; Shelley and Swearer, 2015; Benedetti and Morosinotto, 2016; Morese et al., 2018).

In detail:

- Intentionality: Aggressive behavior is guided by the need to overwrite the other to the
- possibility of creating physical harm.
- Systematicity: Bullying becomes persecutory because it manifests itself systematically at every encounter between a victim and a persecutor.
- Asymmetry of power: The victim is unable to defend himself or to react or seek help (Morese et al., 2018).

Although bullying was once considered to be a natural manifestation of aggression experienced by young people linking to a process of growth and maturity, today “[…] it is known as a real social emergency. Bullying comes from a series of factors, such as culture, stereotypes, family, school, social networking, individual characteristics and ways of managing emotions and conflicts […]” (Ivi, p. 101).

Offensive action can be exercised in a variety of ways: through the use of the word (offenses, teasing, threats), by resorting to physical force and contact (in these cases, it is referred to as direct bullying), talking badly about him/her with other comrades (indirect bullying) or excluding the victim from the group using social pain caused by social exclusion (Eisenberg et al., 2003; Eisenberger et al., 2010). Bully is usually characterized by the use of aggression, which in some cases does not only address mates, but also parents and teachers. It has an impulsive behavior and deficit of empathy for its victims. According to Olweus, at the base of violent behavior there is no tendency to anxiety or poor self-esteem; on the contrary bully often has a positive image of itself (Olweus, 1993, 1997). Passive bullies are those who participate in bullying without actively taking part and usually take on the role of gregarious. Each bully is surrounded by at least two to three peoples who act as supporters (Morese et al., 2018).

The term cyberbullying, instead, refers to those acts of bullying, harassment and using electronic means such as email, chat, blogs, cell phones, social media or any other form of communication attributable to the web. “Cyberbullying is usually operationalized as a kind of bullying understood as peer aggression that is intentional and continuous, and involves an aspect of imbalance of power between a victim and a perpetrator or perpetrators (Tokunaga, 2010). Despite the tool used (f.e., new media), cyberbullying often takes place within a traditional group (e.g., school class). However, cyberspace gives Internet users the opportunity to attack other individuals: people known only from the Internet, celebrities, teachers, totally unknown individuals or whole groups of people. Involvement in such actions brings suffering to those victimized as well as potential negative consequences for the perpetrators” (Pyzalski, 2012, p. 305).

There are different forms of cyberbullying and also the internet form has to be considered a true bullying: sending unpleasant photos, as it actually happens, or sending emails containing offensive material can be much more painful than a punch or a football, even if it does not involve explicit violence or other forms of physical coercion. In virtual communities, cyberbullying can also be in a group that, for example, publishes sexual photos shared privately (Pyzalski, 2012). Cyberbullying is often believed to be conducted anonymously–but it is only a popular belief, in fact research shows that only half of cyberbullying is anonymous (Ybarra and Mitchell, 2004).

## Cyberbullying and Digital Technologies

As in other Western countries, also in Italy, according to the Istat survey of 2018, the spread of new technologies among young people is very broad: “85.8% of boys aged between 11 and 17 use mobile phones every day. Seventy two percent of children of that age surf the Internet every day. This share has grown very rapidly from 56.2 to 72.0% over a 4-year period. Girls are the most frequent users of cell phones and networks, 87.5% of whom use cell phones daily and 73.2% access the Internet every day (a percentage that rises to 84.9% focusing on teenagers aged 14–17). Internet access is strongly driven by the spread of smartphones. In fact, only 27.7% of children use the PC every day and this percentage is in sharp decline compared to 40.5 in 2014” (ISTAT, 2018).

In fact, such extensive use of digital media has ended up having an impact on the spread of forms of cyberbullying as well, to the point that cyberbullying has affected 22.2% of all bullied victims. “In 5.9% of cases, actions were repeated (several times a month). The greater propensity of girls/adolescents that use mobile phones and that connect to Internet probably exposes them more to the risks of the network and new communication tools. In fact, between 11 and 17 years there is a higher percentage of victims: 7.1% of the girls who connect to the Internet or have a mobile phone have been subject to constant harassment through the Internet or mobile phone, against 4, 6% of boys. There is also a greater risk for young people than for teenagers. About 7% of children aged 11–13 were bullied through mobile phone or Internet once or several times a month, while the percentage drops to 5.2% among children aged 14–17 years” (ISTAT, 2018).

The development of sites for sharing files, such as videos (all the social media support images and videos), represents another side of the coin: although on the one hand in these sites we find information, reviews of various products and entertainment, on the other they give a significant contribution in strengthening the phenomenon of cyberbullying, at least in its first phase (before the new policy of exclusion of videos that have as their object violent actions).

The psychological consequences and repercussions of the phenomenon, as we will see later in this contribution, are similar to those of traditional bullying; therefore there could be an intense subjective level of suffering that affects the individual and relational area of the victims with serious effects on self-esteem, on socio-affective abilities, on the sense of self-efficacy, on personal identity, anxiety, depression and, in more extreme cases, suicidal ideas can also occur. It is reasonable to believe that the consequences may be even more serious due to the media strength of messages, photos and videos transmitted online or on the mobile phone. Therefore, it is important to think in terms of prevention to avoid having to deal with much more complex and problematic aspects: good information and communication carried out by the main educational agencies, by the family, the school and other educational institutions, can prove to be very useful; in fact it is often misinformation, the policy of silence and the erroneous conviction of not being able to denounce the facts, to ensure that the attackers act driven by the possibility of not being caught and that the victims suffer feeling shameful and wrong. This triggers a dangerous vicious circle that tends to perpetuate itself with the contribution of all social actors.

It might be useful to dwell on some aspects that have emerged in the last decade, namely a series of effects deriving from cyberbullying. That's why we chose to investigate topics such as Flame, Harassment, Denigration, Imitation, Outing, Deception, Exclusion and Cyberstalking. Behind these high-sounding names there are everyday situations that could happen to any boy/girl today; Cyberbashing or Happy Slapping, for example, is a form of cyberbullying that occurs when the victim is hit and assaulted in front of a group of people filming the episode with the phone and then disclosing it and commenting on it. This means that a boy or a group of boys beats or slaps a peer, while others resume aggression with the phone. Furthermore, as Watzlawick et al. (1971) had already pointed out, communication between individuals may also involve harassing content: in the case of cyberbullying it consists of rude, offensive, disturbing messages, which are repeatedly sent over time, by unwanted e-mail, SMS, MMS, and silent calls. Unlike what happens in flames, the properties of persistence (aggressive behavior repeats over time) and the asymmetry of power between the cyber bully (or cyber bullies) and the victim are recognizable here. Cyberstalking occurs when harassment becomes particularly insistent and intimidating and the victim begins to fear for his or her physical security. The offensive behavior is called cyber-persecution. Denigration is the goal of cyberbully.

Without going into the details of the relationship between bullying, cyberbullying and juvenile crime (see Pisano and Saturno, 2008), characterized, for their complexity, by uncertain and confused borders, we limit ourselves to ascertain the possibility that these categories may have overlapping areas and to focus our attention exclusively on the differences between “off-line bullying” and “on-line bullying.” These categories present numerous areas of divergence, as Willard points out in his work “Cyberbullying and cyber threats: responding to the challenge of online social aggression, threats and anguish” (Willard, 2007). In fact, while bullies are students, classmates or schoolmates, cyberbullies can also be anonymous, so that no one knows their identity; while bullying generally remain in the school space, cyberbullying can be spread all over the world; while in bullying it is easy to find a medium disinhibition caused by the dynamics of the class group and the mechanisms of moral disengagement (Bandura, 1986, 1990; Bacchini, 1998; Sutton et al., 2010), there is a high disinhibition in cyberbullying: cyberbullies tend to do online what they wouldn't do in real life. Furthermore, while in bullying, the need to dominate in interpersonal relationships is linked to the inevitable visibility of the bully, to his popularity, cyberpower can use the alleged invisibility to express power and dominance in the same way (Ybarra and Mitchell, 2004). But what seems even more significant is that while in bullying we find a presence of tangible feedback from the victim to which the bully does not pay enough attention, in cyberbullying, the lack of tangible feedback on his action– “I can't see you!” -can hinder more empathic understanding of the victim's suffering (Fonzi, 1999). In this sense, while in bullying it is easy to find deresponsibility (underlined by terms/justifications such as “We are joking,” “It is not my fault”), in cyberbullying it is possible to detect also depersonalization processes: the consequences of the actions can be, in fact, attributed to “Personas” or “avatar” (virtual alter ego) created. In terms of social dynamics, while in bullying, only the bully, the wing and the bully victim (provocative victim) act as bullying, in cyberbullying, anyone, even those who are victims in real life or have low social power, could become a cyberbully (Ybarra and Mitchell, 2004).

## Individualization Processes, Networks and Social Capital. For A Sociological Approach to Cyberbullying

The emerging of socially strongly remarkable phenomena such those connected to cyberbullying (Hinduja and Patchin, 2009) makes especially binding choosing a multidisciplinary approach, thinking that in this way we could understand more deeply its social implications, as well as its impact on the collective dimension of the action (Shariff, 2008). For scholars, this is a stirring challenge, partly because it gives them the opportunity to overcome the strictness of some disciplinary fences that, in the past, had confined researchers' comparison into often too narrow precincts. Therefore, the variety of scientific profiles of the authors of this article allows them to address the issue of cyberbullying from different perspectives, combining the sensitivity of social sciences with the cognitive approach of neuroscience.

As to sociology, the contribution it can offer to the analysis of this phenomenon must start, in our opinion, from the examination of the wider social context transformation, highlighting how the process of progressive individualization which has influenced contemporary society aided to modify the very features of the social capital on which subjects can rely on. If as, among others, Bourdieu (1986) points out, social capital is the product of social relations, it appears even more precious just in the light of the progressive process of deinstitutionalization of the subjects' life trajectories, since it allows them to root their own life project in a common and shared feeling. Indeed, it is precisely when individual biographical paths become uncertain and differentiated that social capital seems to be a strategic resource, since it offers social actors those relational skills through which binding a network of significant relationships. In all respects, these are resources that individuals and/or groups are able to activate by virtue of inclusion in peculiar relational networks, both formal and informal, implicitly promoting the social recognition dynamics. In a micro level, social capital can prove to be an tool fit for protecting the subject–at least partially–from isolation and/or from the risks of today society (Beck, 1992): through the interaction with the nodes that make up his/her network, in fact, social actor can reactivate some mechanisms of social belonging that the crisis of the collective sources of meaning has questioned step by step (Lyotard, 1984).

In his ponderous reflection on the changes which are taking place in contemporary society, Putnam (2000) aims to analyze the consequences of the decrease in social capital in the United States beginning from the 1970's, exploiting a series of indicators such as the crisis in electoral participation and civic commitment, the decline in membership at associations and unions, the decrease in volunteering and so on. The scholar identifies two different forms of social capital, the bonding social capital, which is the result of relationships characterized by a strong and intense emotional bond (such as the one born, for example, among family members, among close friends or in small local communities) and the bridging social capital, typical, instead, of looser and more scattered relationships which, however, can prove strategically profitable, because they enable the actors the access to a large number of social and/or professional networks. As Manago and Vaughn point out (Manago and Vaughn, 2015, p. 193), “the development of bridging social capital […] reflects a more instrumental form of social relatedness that emphasizes the autonomy of the individual within a diverse network of loose ties.” Based on Granovetter (1983) analysis on the strength of weak ties and on the distinction between strong and weak ties, Putnam notes that the bonding capital aims at strengthening the already existing intense community bonds with a potential closing effect toward those individuals not already fitted in the network; the bridging ties, on the other hand, appear to offer social actors a kind of openness to the outside, enabling them a contact with wider and more diversified social networks, so as to facilitate any interaction with new subjects. It should be stressed, however, that social actors can resort to bridging relationships from a purely instrumental and utilitarian perspective, thus exploiting weak social ties in order to achieve specific objectives. In line with this approach, Bauman (2000) highlights how today even interpersonal relations seem to be subject to the typical dynamics of consumer society, where subjects are committed to immediately discarding the relationships from which they neither benefit nor enjoy. This reflection closely recalls that concept of “pure relationship” developed by Giddens (1991) to highlight how, in radicalized modernity, social actors privilege individual autonomy and freedom of choice criteria even within the management of most intimate bonds.

The growing diversity of life trajectories and feasible experiences that accompany the achievement of the individualization process weakens social bonds strength and the perception of the existence of a common destiny of belonging. In such perspective, the very nature of social ties changes, they become more and more provisional and uncertain: “any dense and tight network of social bonds, and particularly a territorially rooted tight network, is an obstacle to be cleared out of the way. Global powers are bent on dismantling such networks for the sake of their continuous and growing fluidity [….]. And it is the falling apart, the friability, the brittleness, the transience, the until-further-noticeness of human bonds and networks which allow these powers to do their job” (Bauman, 2000, p. 14).

However, social capital can also be read from a relational perspective, thus regarding it as a quality of social relations and not as an attribute of individuals or structures. In such a perspective, Donati (2011) calls relational goods as intangible goods, produced and used together by the subjects participating in the relationship, which can't be available outside these conditions of production. For the scholar, therefore, social capital cannot be understood either through an individualistic semantics (close to the conception expressed, among others, by Bourdieu), or through a holistic paradigm (in line with Putnam's reflection who interprets it as a product of social structures), but rather by virtue of a relational approach, which makes it a property of social relations networks.

Anyway, the individualization process modifies these mechanisms, resulting in the creation of increasingly personal and diversified biographical paths, of dynamics that see the centrality of the choices of the subjects emerging at the expense of the role of those norms and regulatory institutions typical of solid modernity. The liquidity of social relations reveals, as a consequence, their fragility, since the subjects would no longer find the required protection and security to activate the mechanisms of belonging and social recognition on which past societies went by. In fact, the very idea of community is being questioned, just as the mechanism of reproduction of that social capital able to trigger the virtuous circuit of trust, of empathy and sharing appears to be blocked. If, as Wellman (2001) points out, in the individualized society the network is the form through which the social experience is structured, it is clear that also the community dimension loses ground compared to the creation of personal networks centered on the individual and his/her needs. In this perspective, social actors define their membership in a revocable and instrumental way, diversifying their own emotional investment among the networks to which they temporarily choose to join. Adopting the networked individualism paradigm (Rainie and Wellman, 2012) means, therefore, freeing the action of subjects from the dynamics of identification in a single group or in a community, in order to insert them, instead, into a new digital environment within which the individual is the fulcrum of social relations. In fact, networks created through such interaction modes are characterized by multiple and temporary memberships, looking more like networks of individuals connected for specific practical and/or emotional needs, than like integrated groups of subjects oriented to the build of a common project. What is missing, in some respects, is that collective dimension of the action that characterized traditional communities, where the level of internal cohesion was much higher than today.

Acting within a more and more fluid social context, subjects create individual paths among different networks, thus integrating in their own social capital a growing number of weak ties. “Within these forms of networked sociability” (Castells, 2006), individuals set all the time new connections, activating, from time to time, those offline and online links that appear more functional to their purposes.

If, in the dynamics of daily interaction, the emphasis is increasingly placed on the autonomy and independence of social actors, the traditional forms of collective organization of existence end up on the margins of public discourse, making room for new digital technologies and platforms, through which staging contemporary sociality. Already several years ago Ellison et al. (2011) pointed out how Social Network Sites (SNS), and Facebook in particular, had increased the amount of weak ties at individuals' disposal (and, implicitly their supply of bridging social capital), since these platforms support loose social ties, providing infrastructure for the dissemination of social information and allowing users to build and maintain diffuse networks of relationships from which they could potentially draw resources (see also van Dijck et al., 2018). Also in response to the growing individualization, subjects set new contacts and become part of new social networks, using as well social media as spaces where they pour their need for intimacy (Sennett, 1986). However, as Bauman (2001) points out, relationships created within these “peg-communities” are fragile and ephemeral, “bonds without consequences” (Bauman, 2001, p. 71) the Polish sociologist defines them, relationships that do not bind individuals to any form of long-term commitment. That's why social actors, even showing a strong will of anchors and roots, can't find in such networks that steady response to their need for safety and support which only the solid past communities have been able to offer. According to Bauman, sharing of emotions and feelings determines the creation of aesthetic communities, rather than ethical communities, short-lived aggregations within which individuals participate just in limited and short-term commitments, triggering a sort of revision of the most consolidated social protocols.

But the scholar's analysis goes further, underlining how, within these communities without responsibility, social glue can also be represented by a shared aversion or worry, so as to immediately pour individuals' fears into an apparent hostility toward a common target. In this sense Streeten (2002) signals the existence of a negative social capital, an antisocial capital, able of fueling exclusion and discrimination, instead of promoting integration and social cohesion. In fact, identifying a target to be banned or on which focus the dislike of the online community seems to be one of the mechanisms underlying many forms of verbal aggression and cyberbullying conveyed through the Internet and social media, almost like identifying a common enemy were functional to the strengthening of what Corsten (1999), in a contest of different analysis, defines “we-sense.” Within these low-quality social capital networks relationships seem going by connections lacking in mutual responsibility, giving rise to interactions that do not have a shared symbolic horizon as reference. Lacking in a common project, such scattered communities create temporary and revocable emotional ties, using the network as a tool of self-affirmation, rather than a means of comparison and mutual openness. In this sense, cyberbullying is a systematic abuse of power which occurs through the use of information and communication technologies repeatedly and over time. If social networking mechanisms let social actors to overcome physical and structural constraints, going to define a new public (or semi-public) sphere, dynamics established in the online dimension end up delivering the victim of cyberbullying event to a potentially infinite connected audience, since messages, photos and videos quickly turn into viral contents able of traveling, almost independently, on the net. In this sense, as also Boyd (2014) claims, social media have not altered the dynamics of bullying radically, but have made these dynamics more visible to more people. In her analysis, the scholar underlines how, above all among young people, the practice of online sharing has turned into a sort of current currency that gives social visibility to the subjects, making them immediately popular, even at the expense of peers to whom seemingly they do not seem to show any kind of empathy. Boyd (2014, pp. 143-144) writes: “these technologies also allow people to maintain social ties more easily providing infrastructure for the dissemination of social information […]. At the same time, what is shared and easily accessible is not always beneficial. Because social media makes it easy to share information broadly, people can also easily spread hurtful gossip in an effort to assert status, get attention, or relieve boredom. These dynamics are often intertwined.” In fact, if cyberbullies aim to have an audience in front of which performing and from which getting a sort of social recognition, they appear preferring social media as a favorite place for staging their performances, since the latter stands for a social space within which exhibiting and testing values and modes of behavior–censurable in any other context–seemingly without risking any type of social sanction. Unfortunately, among the most serious consequences of such behaviors, possible doubts about the perception of themselves by the victims can also emerge: accusations and negative comments raised online by cyberbullies may end up stuck in the identity conception of bullied subjects, who feel almost forced to negotiate their own self-representation with the fictitious image built within the network. It is as if, in some way, the bearer of such a digital stigma were called to deal with the viral representation of the self conveyed by the SNS, experiencing almost a sense of helplessness and lack of control with respect to the process of building one's own identity. As Slonje et al. (2012) write, “the impact of cyberbullying is clearly negative, including feelings of anger, fright, depression, and embarrassment.”

## Empathy: A Psychological Model for Understanding Cyberbullying

The ability to understand people's feelings and thoughts is a fundamental aspect of social intelligence and is necessary in the social interactions of everyday life. Singer and Lamm (2009) defined this ability as “human empathy,” as a complex phenomenon composed by sub-skills, sub-components and systems. Currently in cognitive sciences different definitions and models of how the emotions of others are understood coexist.

Singer and Lamm (2009) distinguished empathy from emotional contagion, from the theory of the mind, sympathy or compassion. Emotional contagion: precursor of empathy, it cannot be considered as an empathic response as it does not involve emotions, but simply the physiological reactions congruent to the emotional state expressed by other people (e.g., dilation of the pupil) (Singer and Lamm, 2009). Empathy: Hein and Singer (2008) defined empathy as the emotional state caused by the sharing of emotions and sensory states of other people and the empathic process as an isomorphic affective state caused by the observation or imagination of an emotion experienced by another person and of which one is aware (de Vignemont and Singer, 2006). Theory of the mind: the ability to represent the mental states of others including affective ones (Singer and Lamm, 2009). Sympathy or compassion: ability to feel feelings but which are not necessarily the same as those experienced by another person (Ales Bello, 1999; Singer and Lamm, 2009).

Preston and de Waal (2002) differentiate and define the concepts of emotional contagion, sympathy, empathy, cognitive empathy and pro-social behavior: Emotional contagion, an emotion similar to that perceived is activated in the subject; Sympathy, with this term the authors refer to the concept of compassion. They consider the non-correspondence of the same emotional states between those who observe and those who express an emotion necessary (Preston and de Waal, 2002), this mechanism implies a distinction between one's emotional processes and those of the other. Empathy, it requires that you experience the same type of emotion as the other and that the difference between your emotional states and those of the other is maintained (Preston and de Waal, 2002). Cognitive empathy, the ability to represent the mental states of the other, also due to an accurate perception of the situation and the possible behaviors that may derive from it (Preston and de Waal, 2002). Prosocial behavior, action aimed at helping someone who expresses a situation of malaise (Preston and de Waal, 2002). When only cognitive and not affective empathy is present, higher levels of bullying are observed (Jolliffe and Farrington, 2011). Lack of empathy can cause the development of problematic dysfunctional behaviors, such as bullying and cyberbullying (Morese et al., 2018). Furthermore, high levels of empathy have been shown to be associated with less aggressive and more prosocial behaviors, most likely because associated with a greater ability to regulate one's emotions (Meltzoff and Decety, 2003; Kowalsi and Limber, 2013; Vaillancourt et al., 2013; Meuwese et al., 2015; Faucher, 2018; Morese et al., 2018; Luthar and Pušnik, 2020).

Morese and Longobardi (2020) stressed on the processes of regulating emotions especially in situations of social exclusion such as bullying because they can increase the perception of negative emotions and also lead to suicidal thoughts and suicide in adolescence (Morese and Palermo, 2019). As previously reported, the term empathy usually indicates a complex and multidimensional construct ranging from simple emotional contagion to more sophisticated prosocial behavior, but among the various models described it would be important to conceptualize a broader and more transversal framework.

**Figure 1:**
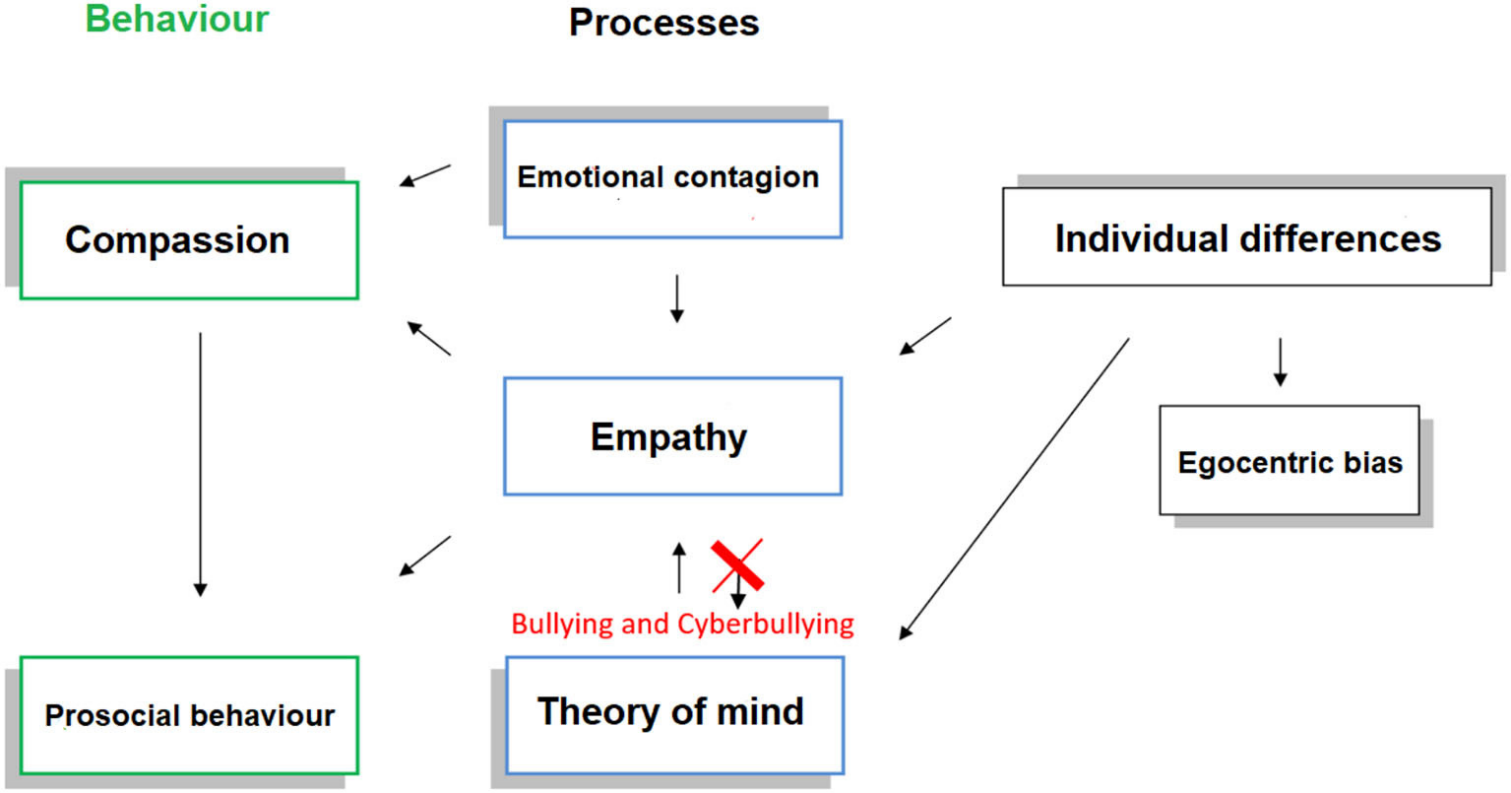
The representation of the model indicates the relationship between the various elements: emotional contagion, empathy, theory of mind, compassion, pro-social behavior, egocentric bias and individual characteristics. Prosocial behavior is the antagonist of cyberbullying, in fact it is possible only by the empathic process that emerges through different processes and influenced by individual indifferences and egocentric bias. The phenomenon of cyberbullying emerges from the breaking point between the theory of mind and empathy.

We proposed in the present theoretical perspective a theoretical model to understand the cyberbullying phenomenon that includes the following elements: emotional contagion, empathy, theory of mind, compassion, prosocial behavior, egocentric bias, and individual characteristics (Figure 1).

Emotional contagion, we applied for the definition of emotional contagion that indicated by Hatfield et al. (2014) according to which it represents the human tendency to synchronize, automatically imitate facial expressions, movements, posture with those expressed by another person. This aspect can represent the most primitive component of empathy. Empathy is the ability to feel the emotions of self and others. Theory of the mind the ability to understand the mental states of self and others oriented useful for predicting behaviors. Compassion. According to Singer and Lamm (2009) compassion represents an emotional state different from that experienced in empathy, more precisely the emotion experienced by the observer does not coincide with that observed, for example the person observes a person who expresses sadness does not experience the same feeling, but that of pity or affection. Both the concept of empathy and prosocial behavior are closely associated with it. Prosocial behavior, according to Chakrabarti and Baron-Cohen (2006) we conceptualize this behavior as oriented toward altruistic action. It represents the way in which the observer feels an emotional response to what the other feels and the desire to relieve suffering, specific to a class of emotions (sadness and pain, but not disgust and happiness) and closely associated with empathy individuals and to theory of mind (Baron-Cohen, 2009). Egocentric bias, the propensity to confuse the mental states of others with one's own as “egocentric bias,” ignoring their possible differences. Individual differences, empathy appears to be influenced by individual differences such as hormonal and genetic (Rodrigues et al., 2009; Collier et al., 2013).

In conclusion, this theoretical model aims to present all aspects associated with the concept of empathy and understand how the empathy element is fundamental to prevent cyberbullying.

All elements within the model are important for the empathic process useful for promoting prosocial behavior. “Empathy” and “Theory of the mind” can be considered two distinct processes, but also connected to each other and influenced by factors such as individual differences. In cyberbullying this does not happen. The breaking point is in the ability to understand the emotions of others but not to feel the emotions of others, therefore without empathy but only theory of the mind. The empathy element is fundamental to prevent cyberbullying and to promote prosocial behavior.

## Absence of Empathy? Cyberbullying in the Age of Hyperconnection

Today we have to consider cyberbullying as a wider social complex. It is necessary starting from the logic of exclusion that Bigo (2008) underlined in 2008 in “Terror, insecurity and liberty.” In fact, we live more and more often in a thin line excluded/who excludes (the ancient victim/executioner dichotomy is evolving today). Trying to amplify, but at the same time simplify, this dichotomy, we can say that today we live in the era of the so-called ban-opticon, or the logic of the ban, which goes from the simple exclusion of friendship on the net (Facebook) to exclusion in a video game (perhaps within the already restricted circle of PlayStation friendship). What catches our attention is that today, just as in 1642 (the reference goes in particular to a novel, The Scarlet Letter) there is a constant, that is, the public pillory as an expiation of “sin,” in this case the adultery. This brutal mechanism continues even now, when society needs to lash out against someone to regenerate and feel united. The mechanism is similar to what we have read before: the chosen person who becomes, for a longer or shorter period, “the monster.” A trademark, a label is imposed on it, just like the letter A of the novel and the community process proceeds before the legal one. We could almost say that bullying, understood as a mental act deriving from a label, has always existed. This is the production mechanism that transforms us into goods, labeled and ready for consumption, an increasingly immediate and faster consumption that becomes viral with the advent of new technologies (Bigo, 2008). In this regard and according to Howard S. Becker as he points out in “Outsiders” (1963), the victims of the labeling would be above all those who commit crimes, which generate social alarm and do not have adequate, material and immaterial means (such as high social status), to counter this label. Consequently, the very definition of the status of “labeled” would be influenced by those who expose the social denunciation of a certain behavior, resulting more effective in those who are on a higher step of the social scale. The follows is that the social reaction is not activated in the same way for all types of crime, resulting more serious against the micro crimes and crimes attributed to minorities, causing less clamor for the crimes originating from the so-called white-collar workers (Becker, 1963). Consequently, there is also an online exhibition which, through the virality of the content, activates online labeling. The latter goes beyond the simple medieval public pillory, since that was a community (usually small urban realities), which fully reflects Becker's theory. In fact, let's see how the protagonist of the novel, Hester, decides to flee to start a new life. Today this escape, to start again, is no longer possible. Viral labeling and, therefore, the transition to cyberbullying, goes beyond the community and the limits. Communities change and evolve and consequently their internal apparatus (actions and interactions) evolves.

Making a brief and rapid historical excursus, we see how the communities, previously limited, were characterized by a rapid, direct and in some way merciless interaction. The slightest transgression of social rules would have led to what we find in “The Scarlet Letter,” that is, the public pillory. As just said, communities evolve and the first evolution took place with the first effects of globalization, in the so-called pre-web communities, where the interaction within them began to change; the symbol of that period was the man called “flaneur,” the one who loved to walk and his emotions were endless. Before proceeding with web society, it is right to call upon Goffman (1959), an author who will be useful to understand the daily life that characterized pre-web communities and to explain how in the web society, so distant, but at the same time similar, there has been a return of community regulations, in certain aspects medievals. Goffman in his sociology of daily life, described in the text “The Presentation of Self in Everyday Life” (1959), analyzes minutely the social interactions in the communities using the dramaturgical metaphor; in fact we find the actor who is always willing to enter the scene, on a stage and in front of an audience (obviously without a fixed screenplay). His idea is that social groups fall into two categories: show groups and audience groups (just like in a theater show). To summarize his thinking, we could argue that social life is a representation according to which groups are staged in front of other groups and everything falls within the community or communities dynamics. Obviously, we find a background, hidden from the public (the example that Goffman uses is that of the hotel waiters), in which private behavior could contradict the public behavior. So, according to Goffman, social life is based on the delimitation of the boundaries between stage and backstage and, consequently, social interaction is a drama that takes place on a scene, in which the actors try to have a control (through impressions management), in order to present themselves in the best possible conditions and in a credible way. Also the groups of spectators have their own structure and behaviors just like in a theater, for example the mask, the companion, the pure spectator or other elements that we find in a theatrical representation. A final element, fundamental in Goffman, is the Self (self-awareness) which is conceived as a contingent element established by the situation, by the stage on which it is performed and by the spectators watching the show (Goffman, 1959).

Today in web society we notice the presence of these elements expressed by Goffman and we see that the public pillory has come to the rescue in a stronger and more cruel way. Indeed, virality is the element of the greatest social contagion. To bring a practical example of what we wrote between Becker and Goffman, we can talk about suicides and homicides against those who made that content viral. Acts that bring current society back to an immediate confrontation with medieval Puritan society. The clear example is that of the stage located in the center of the country and on it a condemned man, an executioner and the community that assists and decides to kill him for purification from the sin committed. Today the mechanism is identical, even if two elements come into play, the first is what has just been described, the second is the absence of empathy on the net. First of all, to paraphrase Goffman, our representation undergoes an update. In fact, the network takes the place of the stage, the executioner is intrinsic in us (we will return in a moment) and the decision of the community is fundamental in a postmodern society, which generates notoriety through virality. A notoriety that can be positive if you are aware that you want to please in a certain way, but it can be negative (and therefore subject to cyberbullying) in the event that the awareness of the subject is to become involved but not memorable. In the latter case, the public mechanism of the pillory is triggered just like in Hawthorne's novel, but with worse consequences. In fact, the audience that assists and consequently makes the content viral chooses to mock a person by exposing them to bullying (Angrove, 2015). This will trigger what we have called the intrinsic executioner. We become executioners of ourselves, we reach extreme acts of liberation (the medieval atonement from sin) precisely because we are no longer allowed to escape from the community (the world is the new viral community); we will not feel able to start a new life anywhere. We must consider empathy as the ability to put yourself in another person's situation or, more precisely, to immediately understand the other's emotional processes. This term is intended to explain a German term, Einfühlung (Treccani, 2019a). The latter indicates what we generally call “identification,” that is, the ability to establish an emotional relationship with people, things, environments, situations and animals. Another very important element is Verstehen (Schutz, 1932; Treccani, 2019b). First, it has been used in the context of German philosophy and social sciences in general since the end of the nineteenth century with the particular sense of interpretative or participatory examination of social phenomena. The term is closely associated with the work of the German sociologist Max Weber, whose anti-positivism, described in Weber (1922), established an alternative to previous sociological positivism and economic determinism, rooted in the analysis of the action corporate. In anthropology, Verstehen means “a systematic interpretative process in which an external observer of a culture tries to relate to it and understand others” (Weber, 1922). It is also seen by Weber as a central concept and a method of rejecting positivist social sciences. Basically, it refers to understanding the meaning of the action from the actor's point of view. We enter the shoes of the other and therefore we treat the actor as a subject rather than an object to be observed. It should be emphasized that the sociology of interpretation (Verstehende Soziologie) is the study of society that focuses on the meanings that people associate with their social world (Weber, 1922).

It would seem that what has just been described is missing from the net and empathy does not find its place because of the “cold” medium that allows us to interact. But, and about that, a definition of online and offline community is given by one of the main sociologists who study these dynamics, namely Barry Wellman in the text “Networks in the Global Village” (1999). He says that virtual communities should not be opposed to physical ones, since they have their own rules and dynamics. The increasing interaction and interdependence between real and virtual contributes to create, for the individual, a new social environment, characterized by belonging to multiple networks of relationships, which determine the birth of each person's “personal communities,” that is, social networks characterized by informal interpersonal bonds, in which the Internet and multimedia profoundly modify the social interaction between the same individuals and between online and offline communities (Whytt, 1765; Wellman, 1999). We could imagine an Internet divided into three parts, each of which has subtle logics of virality. In fact, we find the excluded, the marginal, the most exposed to being victims of intimidating acts (online bullying). It should not exist in a world born as free, but it has become the Panopticon for excellence. The second part is reserved to negative virality, to those who are made “negatively famous” because of the trivialization of the body. Finally, we find those who manage to exploit virality to their advantage by making themselves “positively famous.” Of course, the structure of the Internet community is not so simple, there are exhausting logics that can't be described within the space limits of this essay. Simplifying, virality could look like this three-part scheme. We could conclude that the excluded, the labeled, the mistreated suffer a worse viral return than those who are made negatively famous; this is because, according to Puritan logic, the excluded are those who must face the sneers of the strong community. It is part of the logic of the tag (Facebook/Instagram/Twitter, for example, which allows you to tag people in its content). Therefore, a member of the community questions a topic to make him view the content, to engage him. Right here the virality of the return toward the excluded takes place, in order to have two cases: the first is that the excluded will continue to be excluded because they will not receive the tag; the second, more cruel, sees the excluded person receiving tag on the content useful for deriding him and making him aware of the fact that he is and will always be excluded. So, the brutality of the labeling passes through the brutality of the network (Auriemma, 2017). The approach to a novel set in 1642 with the life of 2020 concerns two crucial aspects, on the one hand a cultural parallelism that in some aspects has not been overcome or improved, in reality it has remained unchanged even if it has evolved in the concept; on the other hand, the novel's ability to get out of writing, become real and give lifeblood to societies. Let us dwell on the first point. We could argue that the Internet, understood as a community square, shares some internal rules of a medieval community, such as labeling and the consequent exclusion from public life, or at least part of it. This is an internal regulation that leaves no room for cultural improvements. We see new techniques for networking, which basically tend to exclude sections of the community, classifying them as unsuitable for what is created (Auriemma, 2017). What leads the reader to reflect on these topics, gives a soul to the text and encloses it in the social body. We could think of the minuteness of the details thanks to which Hawthorne manages to take us away from one world (the current one) to enter another (the world of the mid 1600's), but at the same time he manages to give the text a body and a soul that is the body of today's society. By reading the first pages we are able to guarantee a present and a future for the novel. Above all we are able to extrapolate it from one place and “install” it to another one (Auriemma, 2017). Thanks to our mind we proceed to virtual transfers that allow us to change the structure of the world. Cooperation is increasingly distant from community life, tending above all to the struggle for primacy, where someone (the first) takes everything. A kind of struggle for survival. Where there is the struggle to seize the future that precedes us, to become the best, but at the end of the race we will not know what to do with our isolated success.

## Conclusion

To conclude, we can safely say that there are still many actions to be taken to stem these phenomena. What seems worrying is that traditional socialization agencies, school and family first of all, do not seem sufficiently equipped to deal with cyberbullying, often overlooking the value of the psychological, relational and communicative skills needed to manage the rules of social interaction; moreover, the pervasiveness of digital media makes the established mechanisms of social attention and surveillance rapidly obsolete, keeping also in mind that the element of empathy is fundamental to prevent cyberbullying and promote prosocial behavior. For example, aiming at the birth of initiatives to foster socialization and the use of new technologies would be an important step to explain and analyze the role of the network. In this way we can educate people to use the Internet first and then social networks. For this reason, we believe that scholars, the media and institutions must reflect on the social skills needed to interact today in new digital contexts, promoting a more careful reflection on the impact of social transformations and on the life paths of the younger generations.

## Author Contributions

VA, RM, GI, and GR conceptualized the contribution, wrote the paper, reviewed the manuscript, and provided the critical revision processes as PI. All authors approved the submission of the manuscript.

## Conflict of Interest

The authors declare that the research was conducted in the absence of any commercial or financial relationships that could be construed as a potential conflict of interest.
